# USP34 Haploinsufficiency as a Cause of Neurodevelopmental Phenotypes

**DOI:** 10.1111/cge.70194

**Published:** 2026-06-18

**Authors:** Helena Wigoda, Amjad Khan, Bryce A. Mendelsohn, Noriko Miyake, Nobuhiko Okamoto, Naomichi Matsumoto, Patricia J. C. Knijnenburg, Johanna M. van Hagen, Jiddeke van de Kamp, Quinten Waisfisz, Bryn D. Webb

**Affiliations:** ^1^ Department of Pediatrics Center for Precision Medicine University of Wisconsin School of Medicine and Public Health Madison Wisconsin USA; ^2^ Department of Zoology Institute of Biological Sciences, University of Lakki Marwat Lakki Marwat Khyber Pakhtunkhwa Pakistan; ^3^ Department of Medical Genetics Kaiser Permanente Oakland Medical Center Oakland California USA; ^4^ Department of Pediatrics Nagasaki University Institute of Biomedical Sciences Nagasaki Japan; ^5^ Department of Medical Genetics Osaka Women's and Children's Hospital Izumi Osaka Japan; ^6^ Department of Human Genetics Graduate School of Medicine, Yokohama City University Yokohama Japan; ^7^ Department of Human Genetics Amsterdam UMC Amsterdam the Netherlands

**Keywords:** 2p15p16.1 microdeletion syndrome, autism spectrum disorder, developmental delay, neurodevelopmental disorder, USP34

## Abstract

The 2p15p16.1 microdeletion syndrome is a rare neurodevelopmental disorder caused by heterozygous deletions of variable size involving multiple dosage‐sensitive genes. Within the narrowed critical interval, *USP34* has emerged as a particularly strong candidate for the core phenotype, supported by reports of smaller deletions involving only *USP34* and *XPO1*, and by the fact that *USP34* encodes a deubiquitinating enzyme that stabilizes Axin and thereby regulates canonical Wnt/β‐catenin signaling, a pathway critical for neurodevelopment, craniofacial morphogenesis, and limb patterning. We report six individuals with heterozygous loss‐of‐function variants in *USP34*, including five with confirmed de novo variants, associated with global developmental delay, craniofacial dysmorphism, marked speech impairment, variable autism spectrum disorder, and distal limb anomalies. The phenotype associated with isolated loss of *USP34* overlaps substantially with that reported in 2p15p16.1 microdeletion syndrome, while suggesting that some features seen in larger deletions may reflect the contribution of additional genes within the interval. These findings support haploinsufficiency of USP34 as sufficient to cause a distinct neurodevelopmental disorder, establish *USP34* as a major contributor to the neurodevelopmental and dysmorphic phenotype associated with the 2p15p16.1 locus, and refine gene‐specific contributions within this microdeletion syndrome.

## Graphical abstract

Heterozygous loss‐of‐function variants in *USP34* cause a novel neurodevelopmental disorder characterized by global developmental delay, speech impairment, autism, hypotonia, craniofacial dysmorphism, and distal limb anomalies. Disrupted Wnt/β‐catenin signaling via reduced Axin stabilization refines gene‐specific contributions within 2p15p16.1 microdeletion syndrome.

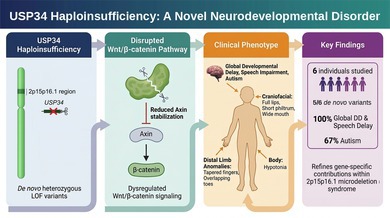

## 1 Introduction

2p15p16.1 microdeletion syndrome is a rare neurodevelopmental disorder arising from heterozygous deletions at chromosome 2p15p16.1 that vary considerably in size, and may include loss of the genes *XPO1*, *USP34*, *BCL11A*, *REL*, *PAPOLG*, *PEX13*, *COMMD1*, *B3GNT2*, and *EHBP1*. Despite the genomic heterogeneity, the syndrome is defined by a consistent core phenotype of global developmental delay and moderate‐to‐severe intellectual disability in virtually all affected individuals [1, 2]. Associated features, including microcephaly, hypotonia, autism spectrum disorder, structural brain abnormalities, craniofacial dysmorphism, hearing loss, intrauterine growth restriction, short stature, and digital anomalies, are each reported in a substantial proportion of patients but are not universally present, and this phenotypic variability has made precise genotype–phenotype correlations difficult to establish. The broad phenotypic spectrum suggests that the syndrome results from the combined dosage‐sensitive effects of multiple genes within the deleted interval rather than from a single unifying pathogenic mechanism [2, 3]. This is further supported by the recognition of monogenic neurodevelopmental disorders caused by haploinsufficiency of neighboring genes within this interval, including *BCL11A* and *XPO1* [4, 5]. More restricted de novo deletions of approximately 103 to 238 kb, encompassing *USP34* and *XPO1*, have been reported in patients with developmental delay, intellectual disability, language delay, hypotonia, craniofacial dysmorphism, abnormalities of head growth or morphology, high‐arched palate, digital anomalies, and structural brain abnormalities, such as corpus callosum anomalies and Chiari malformation [1, 6, 7, 8]. Systematic candidate gene analyses and trait‐driven statistical modeling across cohorts with 2p15p16.1 microdeletions identified *USP34* and/or *XPO1* as associated with hearing loss, microcephaly, and intrauterine growth restriction [3].

Among the genes within the deletion, *USP34* is a particularly compelling candidate for contributing to the neurodevelopmental phenotype. *USP34* encodes a large ubiquitin‐specific protease that was identified by mass spectrometry as a component of the Axin destruction complex and shown to stabilize Axin by opposing tankyrase‐mediated ubiquitination and proteasomal degradation, thereby acting as a positive regulator of canonical Wnt/β‐catenin signaling [9, 10]. Axin itself is a multifunctional scaffold protein whose regulation by post‐translational modification, including ubiquitination, phosphorylation, and poly‐ADP‐ribosylation, is central to the dynamic control of Wnt pathway activity [9]. Canonical Wnt/β‐catenin signaling plays essential and temporally regulated roles in neural progenitor proliferation, cortical neurogenesis, axon guidance, and synaptogenesis, and dysregulation of this pathway is increasingly recognized as a mechanism underlying a broad spectrum of neurodevelopmental disorders [9, 11, 12]. Consistent with a critical dosage‐sensitive role in development, *USP34* has a probability of loss‐of‐function intolerance (pLI) score of 1.0 and a loss‐of‐function observed/expected upper bound fraction (LOEUF) score of 0.19, indicating that heterozygous loss‐of‐function variants are extremely poorly tolerated in the general population [13]. Haploinsufficiency of USP34 is therefore predicted to reduce Axin stabilization, perturb Wnt/β‐catenin signaling, and impair normal brain development, providing a plausible molecular basis for the neurodevelopmental and structural brain phenotypes observed in affected individuals. Here, we present five individuals harboring de novo single‐nucleotide variants in *USP34* and one individual with a variant not inherited from the only parent available for testing, with all variants predicted to cause loss‐of‐function. We characterize their shared and variable clinical features and compare this cohort with individuals reported with 2p15p16.1 microdeletion syndrome. Our findings establish USP34 haploinsufficiency as the cause of a novel neurodevelopmental disorder and provide a clinical and molecular framework to guide interpretation of *USP34* loss‐of‐function variants identified in diagnostic sequencing.

## 2 Materials and Methods

### 2.1 Human Subjects Research

Cases in this series were identified through GeneMatcher and international collaboration [14]. Research consent was obtained for each participant under protocols approved by the respective institutions or coded/anonymized data are being presented as authorized by the institutional review board of the University of Wisconsin School of Medicine and Public Health (Protocol: 2020–0700). All research was conducted in accordance with the Declaration of Helsinki. Written informed consent was obtained from the participants or their legal guardians for the use and publication of clinical photographs.

### 2.2 Whole Exome Sequencing/ Next Generation Sequencing

**FIGURE 1:**
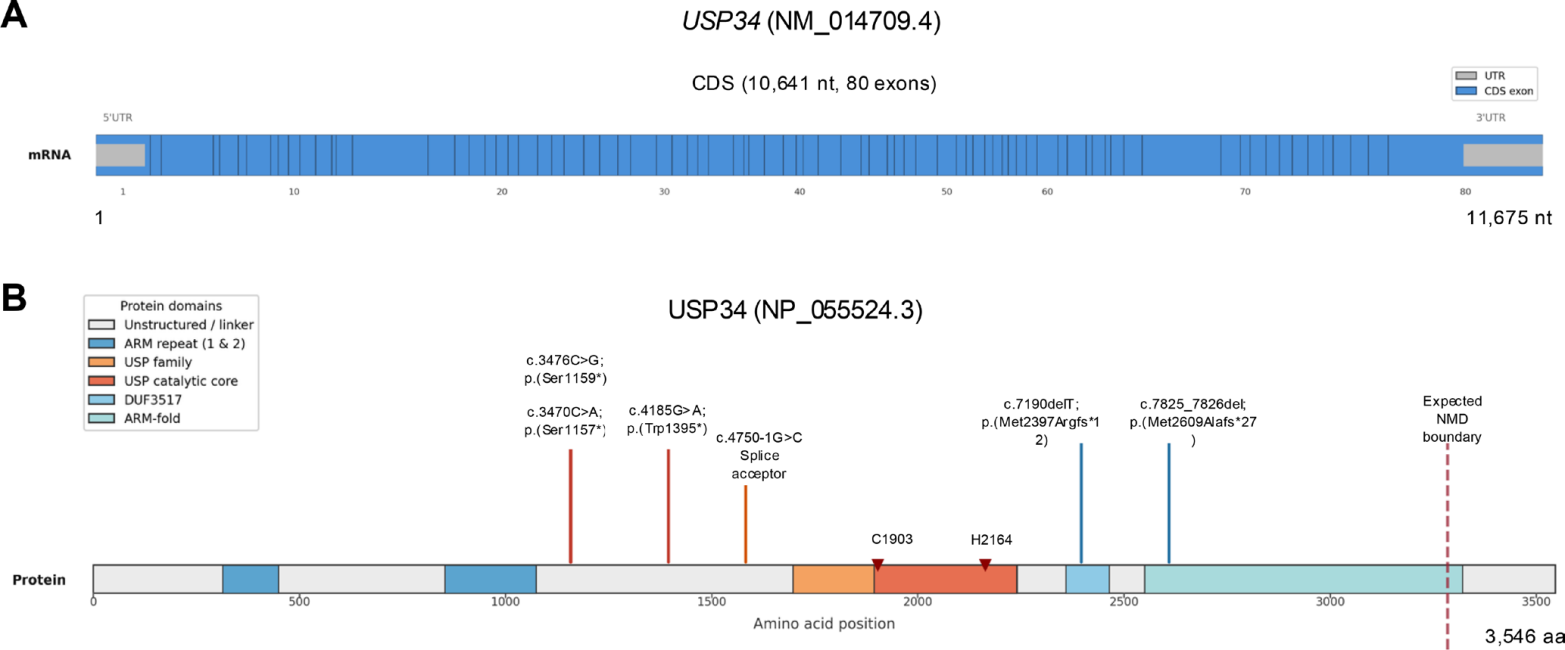
Schematic overview of *USP34* gene and protein architecture with locations of reported variants. (A) Linear representation of the *USP34* mRNA transcript (NM_014709.4; 11,675 nucleotides total). The coding sequence (CDS) is 10,641 nucleotides total (nt 396–11,036) divided across 80 exons (blue bars). The 5′ and 3′ untranslated regions (UTRs) are shown in gray. Exon numbers are indicated below the schematic (1–80). (B) Linear domain architecture of the USP34 protein (3546 amino acids). Protein domains are annotated and include ARM repeat regions 1 and 2 (aa 314–449 and aa 853–1074, respectively); USP family domain; USP catalytic core (aa 1894‐2236); DUF3517 domain (aa 2359–2464); and C‐terminal ARM‐fold/ARM superfamily domain (aa 2550–3321). Red arrowheads (▼) indicate the two conserved active site residues within the USP catalytic core (Cys1903 and His2164). Pathogenic variants are marked. The dashed vertical line marks the last exon‐exon junction (EEJ)/NMD boundary, beyond which premature termination codons are not predicted to trigger NMD. *Abbreviations:* ARM, armadillo repeat; DUF, domain of unknown function; NMD, nonsense‐mediated mRNA decay; USP, ubiquitin‐specific protease.

*USP34* variants were identified by whole‐exome sequencing in Individuals 1–2, 4–6. The *USP34* variant in individual 3 was identified by clinical testing with an expanded autism/intellectual disability gene panel including 2773 genes (Figure 1).

### 2.3 Clinical Data Collection and Phenotyping

Detailed clinical information was obtained through review of medical records and supplemented, when necessary, by follow‐up communication with referring clinicians and families. Phenotypic findings were reviewed by clinical geneticists and genetic counselors to support uniform clinical characterization of the cohort.

## 3 Results and Patient Case Reviews

**FIGURE 2:**
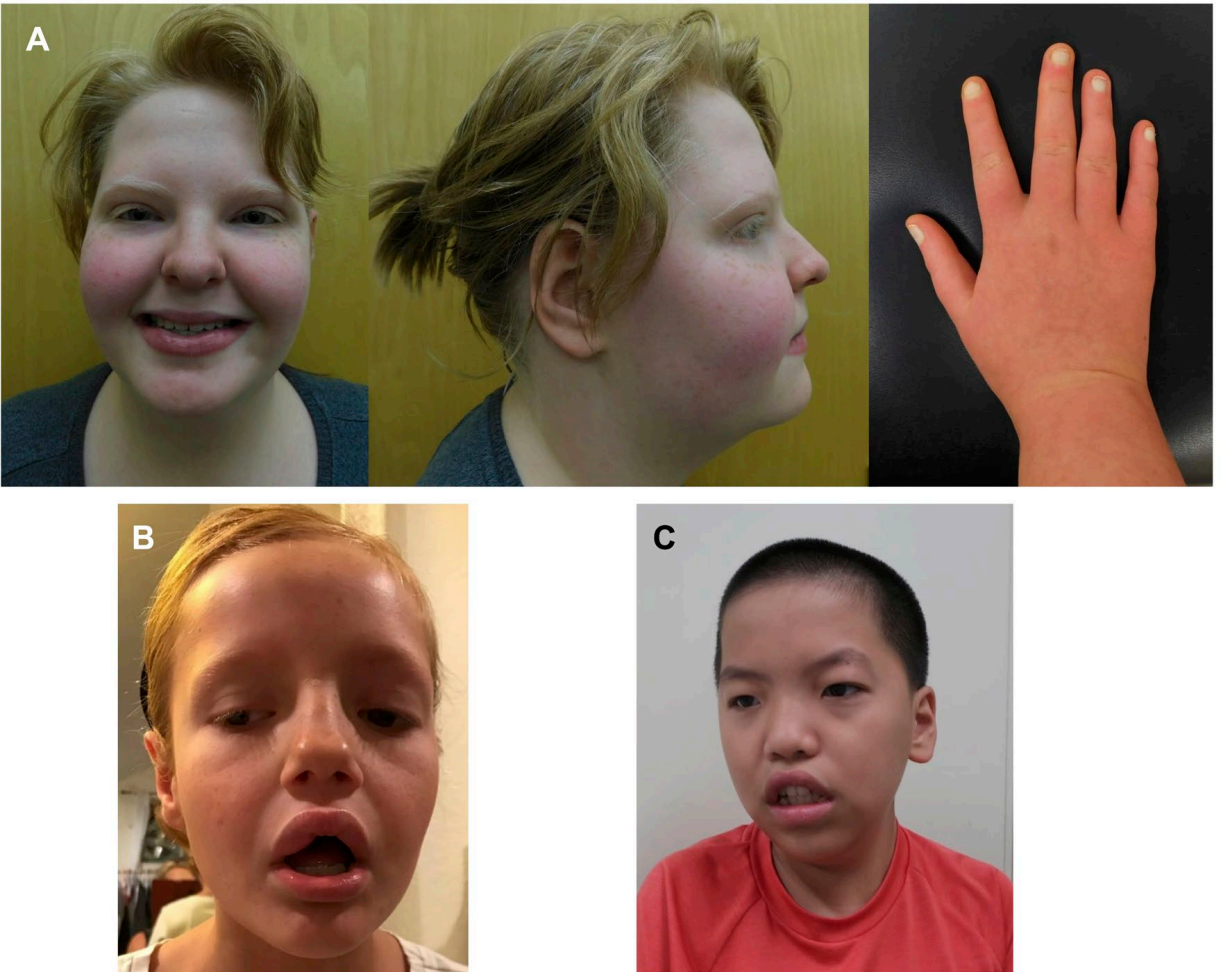
Craniofacial and digital features in USP34 Haploinsufficiency. (A) Photograph of Individual 1 at 14 years old demonstrating deep‐set eyes, large ears, wide mouth, full lips, short philtrum, and tapered fingers. (B) Facial photograph of individual 3 at 9 years old demonstrating deep‐set eyes, downslanting palpebral fissures, wide mouth, full lips, short philtrum, and micrognathia. (C) Facial photograph of Individual 4 at 13 years old demonstrating short philtrum, full lips, wide mouth, and micrognathia.

Individual 1 is a female of mixed northern European and Native American ancestry identified to have a de novo heterozygous nonsense *USP34* variant NM_014709.4:c.3470C>A; p.(Ser1157*) by clinical whole‐exome sequencing (Tables 1 and 2, Figure 2). Karyotype and chromosomal microarray were normal. She was born at 37 weeks and 1 day of gestation to non‐consanguineous parents after a pregnancy complicated by gestational diabetes treated with diet and insulin, and was delivered by Cesarean section for placental abruption, failure to progress, and fetal heart decelerations. Birth weight was 2.58 kg (−0.67 SD), length was 43.2 cm (−2.34 SD), and head circumference was 33 cm (+0.28 SD). Neonatal course was notable for jaundice treated with phototherapy. Upon genetics evaluation at 14 years of age, height was 155 cm (−0.96 SD) and weight was 75.5 kg (+1.71 SD). Head circumference was last assessed at 9 years of age and measured 51.1 cm (−0.65 SD). Her phenotype was characterized by global developmental delay, mild intellectual disability, marked language impairment, attention‐deficit/hyperactivity disorder, oppositional defiant disorder, self‐injurious behavior, and sleep disturbance, without autism spectrum disorder. She also had delayed visual maturation with bilateral astigmatism, strabismus, and long‐standing intermittent horizontal/torsional nystagmus that increases on horizontal gaze and upgaze. Neurologic examination identified nystagmus, hypotonia, broad‐based gait, and dysdiadochokinesia. Brain magnetic resonance imaging demonstrated mild corpus callosum hypoplasia with slight colpocephalic prominence of the lateral ventricles, prominent perivascular spaces, and hypoplasia of the sella. Additional musculoskeletal findings included pes planus and genu valgum.

**TABLE 1:** Genotypic findings and core clinical features of the current USP34 cohort, with comparison to previously reported 2p15p16.1 microdeletion cases.

Feature	Individual 1	Individual 2	Individual 3	Individual 4	Individual 5	Individual 6	Current cohort	Previously reported 2p15p16.1 cases (Miceli et al.; Bagheri et al.)
USP34 variant (NM_014709.4)	c.3470C>A; p.(Ser1157*)	c.4185G>A; p.(Trp1395*)	c.7190delT; p.(Met2397Argfs*12)	c.7825_7826del; p.(Met2609Alafs*27)	c.4750‐1G>C	c.3476C>G; p.(Ser1159*)		
Inheritance	De novo	De novo	Not maternally inherited	De novo	De novo	De novo		
Sex	Female	Female	Female	Male	Male	Male		
IUGR	−	−	−	−	−	+	1/6 (17%)	10/26 (38%)
Age at last evaluation	14 years, 6 months	20 years	16 years	14 years	4 years	3 years, 9 months		
Birth weight	2.58 kg (−0.67 SD)	ND	3.57 kg (+0.79 SD)	3.55 kg (+1.1 SD)	3.82 kg (+0.51 SD)	2.99 kg (−2.3 SD)		
Birth length	43.2 cm (−2.34 SD)	ND	52 cm (+1.57 SD)	51 cm (+1.1 SD)	ND	ND		
HC at birth	33 cm (+0.28 SD)	ND	35.5 cm (+1.33 SD)	35 cm (+1.3 SD)	ND	ND		
Weight at last evaluation	75.5 kg (+1.71 SD)	56.7 kg (−0.16 SD)	60 kg (+0.59 SD)	53.3 kg (+0.4 SD)	20 kg (+1.2 SD)	16.3 kg (+0.28 SD)		
Height at last evaluation	155 cm (−0.96 SD)	139.7 cm (−3.62 SD)	155.6 cm (−1.07 SD)	167.1 cm (+0.2 SD)	109 cm (+0.05 SD)	102 cm (+0.42 SD)		
HC at last evaluation	51.1 cm (measured at 9 years of age; −0.65 SD)	54 cm (−0.28 SD)	55 cm (measured at 12 years of age; +1.40 SD)	59 cm (+3.1 SD)	51.1 cm (+0.05 SD)	ND		
Developmental delay	+	+	+	+	+	+	6/6 (100%)	43/43 (100%)
Global DD	+	+	+	+	+	+	6/6 (100%)	ND
Speech delay	+	+	+	+	+	+	6/6 (100%)	25/25 (100%)
ID	+	ND	−	+	+	ND	3/4 (75%)	28/28 (100%)
ASD	−	+	+	+	−	+	4/6 (67%)	8/19 (42%)
Hypotonia	+	+	+	−	+	−	4/6 (67%)	18/19 (95%)
Structural brain anomalies	+	ND	−	−	−	ND	1/4 (25%)	16/25 (64%)
Hearing loss	−	+	−	+ (sensorineural)	−	−	2/6 (33%)	8/29 (28%)

*Note:* Values are presented as *n*/*N* (%), where *N* indicates the number of individuals assessed for a given feature. Previously reported cases were derived from Miceli et al. and Bagheri et al. Developmental delay was defined as a delay in at least one developmental domain, whereas global developmental delay was defined as a delay in two or more developmental domains.

Abbreviations: +, present; −, absent; ASD, autism spectrum disorder; DD, developmental delay; HC, head circumference; ID, intellectual disability; IUGR, intrauterine growth restriction; ND, not determined.

**TABLE 2:** Dysmorphic features in the current USP34 cohort.

Feature	Individual 1	Individual 2	Individual 3	Individual 4	Individual 5	Individual 6	Current cohort
Downslanting palpebral fissures	−	+	+	−	+	−	3/6
Deep‐set eyes	+	+	+	−	−	−	3/6
Large ears	+	+	−	−	−	−	2/6
Short philtrum	+	+	+	+	−	+/−	5/6
Full lips	+	+	+	+	+	+	6/6
Wide mouth	+	+	+	+	−	−	4/6
Diastema	−	+	−	−	+	+	3/6
Micrognathia	−	+	+	+	−	−	3/6
Digital anomalies	+; tapered fingers; overlapping toes	+; bilateral second–third toe syndactyly	+; long tapered fingers with mild contractures; bilateral index‐finger clinodactyly; long first and second toes	−	+; tapered fingers; mild bilateral fifth‐finger clinodactyly; broad, proximally implanted thumbs	−	4/6
Other dysmorphic features	High hairline, bulbous nasal tip, smooth philtrum	Broad nasal bridge	Bifid nasal tip	Hypertelorism	Hypertelorism	Pectus carinatum	

*Note:* Values are presented as *n*/*N*, where *N* indicates the number of individuals assessed for a given feature. For calculation of the current cohort total, entries recorded as +/− were classified as present.

Abbreviations: +, present; −, absent; +/−, equivocal or mildly present.

Individual 2 is a female of Afghani ancestry with a de novo heterozygous nonsense variant in *USP34*, NM_014709.4:c.4185G>A; p.(Trp1395*), identified by whole‐exome sequencing (Tables 1 and 2). She was born at 38 weeks of gestation to non‐consanguineous parents after an uncomplicated pregnancy. Her history is notable for failure to thrive, developmental delay involving both motor and speech domains, autism spectrum features, behavioral abnormalities, and epilepsy with onset in infancy, treated with carbamazepine. She also had strabismus and reduced visual acuity. Motor milestones were delayed, with independent sitting at 14 months and walking at 19 months. At her most recent evaluation at 20 years of age, she had marked short stature, with a height of 139.7 cm (−3.62 SD), weight of 56.7 kg (−0.16 SD), and head circumference of 54 cm (−0.28 SD). Neurologic examination demonstrated hypotonia involving the shoulder and pelvic girdle, mild dystonia, and gait abnormality, with otherwise normal strength, muscle bulk, reflexes, and sensation. She also had mild hearing loss of unclear etiology with conductive versus sensorineural loss not determined.

Individual 3 is a female of European ancestry with a heterozygous *USP34* frameshift variant, NM_014709.4:c.7190delT;p.(Met2397Argfs*12) (Tables 1 and 2, Figure 2). The variant was absent in her mother; paternal testing was not completed. She was born at 39 weeks of gestation after an uncomplicated pregnancy. Birth weight was 3.57 kg (+0.79 SD), length was 52 cm (+1.57 SD), and head circumference was 35.5 cm (+1.33 SD); there were no perinatal complications. The neonatal course was notable for feeding difficulty that later resolved, and early childhood was notable for recurrent ear infections. Early development was characterized by axial hypotonia, global developmental delay with speech delay, delayed walking at 19 months, and later balance problems. She was found to have borderline intellectual functioning, with a full‐scale IQ of 78, together with autism spectrum disorder, attention‐deficit/hyperactivity disorder, irritability, and aggressive behavior. Later medical history was notable for bilateral myopia, mild scoliosis, and premature thelarche. Brain magnetic resonance imaging at 14 years and 6 months of age showed no clinically significant structural abnormality; minimal inferior displacement of the cerebellar tonsils was noted. Overall growth remained within normal limits, with head circumference at +1.40 SD at 12 years of age, and weight and height at +0.59 SD and −1.07 SD, respectively, at the most recent evaluation at 16 years of age.

Individual 4 is a male of Japanese ancestry with a de novo heterozygous *USP34* frameshift variant, NM_014709.4:c.7825_7826del; p.(Met2609Alafs*27) (Tables 1 and 2, Figure 2). Karyotype was 46, XY and chromosomal microarray was normal. He was born at 39 weeks and 3 days of gestation to non‐consanguineous parents after an unremarkable pregnancy. Birth weight was 3.55 kg (+1.1 SD), length was 51.0 cm (+1.1 SD), and head circumference was 35 cm (+1.3 SD). Early development was notable for global developmental delay with severe speech impairment. Milestones were delayed, with head control achieved at 5 months, sitting at 9 months, and independent walking at 16 months. He was found to have severe intellectual disability, with a full‐scale IQ of 30, together with autism spectrum disorder, hyperphagia, and mild sensorineural hearing loss. At last evaluation at 14 years of age, height and weight were within normal range (+0.2 SD and +0.4 SD, respectively), head was macrocephalic at +3.1 SD. He was able to follow simple commands, but had no meaningful expressive language beyond “yes” and remained essentially nonverbal. Tone, strength, muscle bulk, reflexes, sensation, gait, and coordination were normal. Brain magnetic resonance imaging was normal.

Individual 5 is a male with a de novo heterozygous splice‐site *USP34* variant, NM_014709.4:c.4750‐1G>C (Tables 1 and 2). This variant is predicted to disrupt a canonical splice acceptor site (SpliceAI score of 0.98) [15], consistent with a loss of function mechanism. He was born at 39 weeks and 3 days of gestation to non‐consanguineous parents, with a birth weight of 3.820 kg (+0.51 SD). The perinatal course was notable for clavicular fracture, suspected infection treated with antibiotics, hypoglycemia, and hyperbilirubinemia. Early development was marked by global developmental delay, particularly in motor and speech domains. He stood independently shortly before 2 years of age, walked later than peers, and was described as clumsy, with frequent falls and balance difficulties. By 4 years of age, his speech remained difficult to understand except for by his parents. Additional behavioral features included difficulty with emotional regulation and mild hyperopia. He also experienced a single episode of transient unconsciousness at 4 years of age with hypotonia and temporary inability to walk, although no epilepsy diagnosis was established. Neurologic examination showed hypotonia and no clear ataxia; brain magnetic resonance imaging was normal. At last evaluation at 4 years of age, height, weight, and head circumference were within the normal range (+0.05 SD, +1.2 SD, and +0.05 SD, respectively). Additional findings included diminished abduction of the thumbs.

Individual 6 is a male with a de novo heterozygous nonsense *USP34* variant, NM_014709.4:c.3476C>G; p.(Ser1159*) (Tables 1 and 2). He was born at 40 weeks and 6 days of gestation to non‐consanguineous parents, with a birth weight of 2.99 kg (−2.3 SD), consistent with mild intrauterine growth restriction. Early development was characterized by developmental delay, most notably affecting language; independent walking was achieved at 18 months. He also exhibited autism spectrum features. At his most recent evaluation at 3 years of age and 9 months, he remained nonverbal. Height and weight were within the normal range (+0.42 SD and +0.28 SD, respectively). Neurologic assessment showed persistent gait clumsiness. Additional findings included hyperphagia and thoracic wall anomalies, specifically a broad thorax and pectus carinatum.

## 4 Discussion

We report six individuals harboring predicted loss‐of‐function variants in *USP34* who share a consistent neurodevelopmental phenotype, supporting USP34 haploinsufficiency as the cause of a novel, clinically recognizable disorder. *USP34*‐related neurodevelopmental disorder is characterized by universal developmental delay with marked speech‐language impairment, a high frequency of autism spectrum disorder, motor dysfunction, craniofacial dysmorphism, and digital anomalies. This profile shows substantial overlap with the core features of 2p15p16.1 microdeletion syndrome, while allowing, for the first time, resolution of the *USP34*‐specific contribution from that of neighboring genes within the interval. This comparison is further informed by the recent delineation of two monogenic disorders arising from the same interval, Dias‐Logan syndrome (BCL11A haploinsufficiency) and *XPO1*‐related neurodevelopmental disorder (XPO1 haploinsufficiency), which together provide a framework for dissecting individual gene contributions to the 2p15p16.1 phenotypic spectrum [3, 4, 5].

In this context, *USP34*‐related disease overlaps strongly with the neurodevelopmental phenotype described in 2p15p16.1 microdeletion syndro. Within this shared neurodevelopmental profile, language was especially affected in our cohort, with two individuals remaining nonverbal (2/6), consistent with the marked and recurrent speech involvement reported in both the microdeletion syndrome and in both monogenic disorders involving neighboring genes in the interval [2, 3, 4, 5].

Autism spectrum disorder was highly represented in our cohort, occurring in 4/6 individuals (67%), compared with 42% in previously reported 2p15p16.1 microdeletion cohorts [2, 3]. Prior deletion‐mapping studies and monogenic evidence from Dias‐Logan syndrome have generally pointed more strongly to *BCL11A* than *USP34* as the primary driver of autism within the broader microdeletion syndrome [3, 5]. However, the high frequency of autism spectrum disorder in our cohort indicates that *USP34* loss alone may also be sufficient to contribute to this aspect of the phenotype. The recent description of autism spectrum disorder in *XPO1*‐related neurodevelopmental disorder further supports the view that autistic features within the 2p15p16.1 spectrum do not arise from a single critical locus, but instead reflect overlapping contributions from multiple genes within the interval [4]. Our findings therefore argue that *USP34* is an independent contributor to autism‐related manifestations within the 2p15p16.1 spectrum, even if it is not the sole or primary driver of the phenotype.

Motor dysfunction was a consistent feature of our cohort, and motor delay was documented in all six individuals. Motor delay was universal among those assessed, and gait or coordination abnormalities, including broad‐based gait, imbalance, and clumsiness, were relatively common. By contrast, hypotonia was present in 4/6 individuals (67%), a lower frequency than that reported in published 2p15p16.1 microdeletion cohorts and lower than rates reported in *XPO1*‐related neurodevelopmental disorder [3, 4].

The combination of less frequent hypotonia despite the presence of motor delay suggests that motor dysfunction in isolated *USP34*‐related disease may be expressed predominantly through delayed motor acquisition, coordination deficits, and postural instability rather than generalized hypotonia, and that the high rate of hypotonia in the broader deletion syndrome may reflect contributions from additional genes within the interval.

Abnormalities of head growth and structural brain development were notably less prominent in our cohort than in individuals with 2p15p16.1 microdeletion. Microcephaly was not observed among individuals with available head circumference data, and one individual instead had macrocephaly. This stands in contrast to both neighboring monogenic disorders within the interval as postnatal microcephaly is reported in 50% of individuals with Dias‐Logan syndrome and in 64% of individuals with *XPO1*‐related neurodevelopmental disorder, while abnormal brain MRI findings occur in 59% and 42% of individuals with these respective disorders [4, 5]. The deletion‐based analysis of Miceli et al. associated USP34/XPO1 haploinsufficiency with microcephaly within the 2p15p16.1 interval [3, 16]; however, this analysis could not separate the contribution of *USP34* from that of *XPO1*, and *XPO1* appears more responsible for this feature. The deletion‐based analysis by Miceli et al. also concluded that deletions of *USP34*/*XPO1* are more likely to result in hypoplasia or agenesis of the corpus callosum [3]. In our lone individual with a structural brain abnormality, mild corpus callosum hypoplasia was identified in addition to slight colpocephalic prominence of the lateral ventricles, prominent perivascular spaces, and hypoplasia of the sella. These findings are in contrast to the more extensive abnormalities reported in the broader microdeletion or Dias‐Logan syndrome, including cortical dysplasia, ventriculomegaly, optic nerve hypoplasia, and more substantial callosal or posterior fossa anomalies [3, 16].

Prenatal and postnatal growth also appeared to be affected differently in our cohort. Only one individual in our cohort had intrauterine growth restriction, even though deletion‐based data from Miceli et al. linked IUGR to the shared *USP34/XPO1* interval in 2p15p16.1 microdeletion syndrome. IUGR is present in 10/26 individuals (38%) with 2p15p16.1 microdeletion, while data for IUGR in *XPO1*‐related neurodevelopmental disorder are unavailable [2, 3, 4]. By contrast, short stature, defined as height below −2 SD at last evaluation, was present in 1/6 individuals. Although the small sample size limits firm conclusions, this pattern raises the possibility that abnormalities of both prenatal and postnatal growth may fall within the *USP34*‐related spectrum [3, 16].

Hearing loss is noted in 28% of individuals with 2p15p16.1 microdeletion; however, conductive versus sensorineural hearing loss is not distinguished. In our cohort, hearing loss was present in 2/6 individuals; one had sensorineural hearing loss, whereas in the other the type was not determined. In the deletion‐based analysis by Miceli et al., hearing loss was observed in 8/29 assessed individuals and it was associated with the minimal shared interval with *USP34*/*XPO1* [3, 17]. Hearing loss was not reported among the recurrent features of monogenic *XPO1*‐related disorder, raising the possibility that *USP34* contributes to the auditory phenotype mapped to this shared interval [4]. In our cohort, however, the finding was mild in both affected individuals, and its frequency and clinical significance will need to be clarified in additional patients with isolated USP34 haploinsufficiency [3].

Visual involvement was likewise observed in several individuals, but it was variable. Strabismus was present in two individuals, and other findings included delayed visual maturation, astigmatism, hyperopia, myopia, and nystagmus. By contrast, visual involvement in individuals with 2p15p16.1 microdeletion syndrome typically consists of optic nerve abnormalities when present [2, 3]. The presence of strabismus in two individuals in our cohort raises the possibility of phenotypic overlap with Dias‐Logan syndrome, in which strabismus is also a common feature, reported in 60% of affected individuals [5].

Craniofacial dysmorphism was universal in our cohort, with limited overlap with the facial features reported in the published 2p15p16.1 microdeletion syndrome. Full lips were a universal feature in our cohort, present in 6/6 individuals, while a short philtrum was observed in 5/6, distinguishing the facial phenotype from that of the classic microdeletion syndrome, in which a long or smooth philtrum and thin upper lip are more commonly described. Downslanting palpebral fissures were present in 3/6 individuals and occurred at a similar frequency to that reported in the microdeletion syndrome [2, 3]. Other recurrent features in our cohort included deep‐set eyes in 3/6 individuals, large ears in 2/6, wide mouth in 4/6, diastema in 3/6, and micrognathia in 3/6. Although craniofacial dysmorphism is also recognized in the neighboring monogenic disorders *XPO1*‐related neurodevelopmental disorder and Dias‐Logan syndrome, the overall facial gestalt remains nonspecific [4, 5]. By contrast, our findings suggest a consistent facial gestalt, particularly in the oral region, associated with isolated *USP34* loss, distinct from the more heterogeneous craniofacial phenotype reported in 2p15p16.1 microdeletion syndrome [2, 3].

By contrast, distal limb involvement showed substantial phenotypic overlap with the broader 2p15p16.1 microdeletion phenotype. Digital anomalies were present in 4/6 individuals and included tapered fingers, overlapping toes, second‐third toe syndactyly, mild contractures, clinodactyly, broad thumbs, and elongated first and second toes. The relatively high frequency of distal extremity abnormalities in our cohort is consistent with the microdeletion literature and appears greater than in the monogenic *XPO1*‐related disorder and Dias‐Logan syndrome, suggesting that distal limb manifestations within the 2p15p16.1 spectrum may be more strongly associated with *USP34* loss than with disruption of either neighboring gene alone [4, 5].

## 5 Conclusion

Our findings support isolated USP34 haploinsufficiency as a recognizable neurodevelopmental disorder within the broader 2p15p16.1 spectrum, with substantial phenotypic overlap with both the classic microdeletion syndrome and with the neighboring monogenic disorders caused by *XPO1* and *BCL11A* disruption, while also helping refine gene‐specific contributions within the interval. In our cohort, the most consistent manifestations were global developmental delay, particularly marked speech‐language impairment, and craniofacial dysmorphism, which was present in all individuals in the cohort. Autism spectrum disorder also occurred frequently, indicating that *USP34* contributes to the shared phenotype of 2p15p16.1 microdeletion syndrome. At the same time, the absence of microcephaly among individuals with available head circumference data, together with the relative mildness of structural brain abnormalities, suggests that USP34 haploinsufficiency is unlikely to account for the abnormal head growth and major brain malformations observed in 2p15p16.1 microdeletion syndrome and distinguishes it from the neighboring monogenic disorders caused by pathogenic variants in *XPO1* or *BCL11A*. This pattern supports a model in which 2p15p16.1 microdeletion syndrome reflects overlapping but non‐identical contributions from multiple dosage‐sensitive genes and positions *USP34* as an important contributor to the neurodevelopmental features that characterize the broader phenotypic spectrum associated with 2p15p16.1 microdeletion.

Biological support for these genotype–phenotype inferences comes from the known role of *USP34* in Axin stability and Wnt/β‐catenin signaling, together with experimental evidence for involvement in BMP‐related osteogenic pathways [10, 18, 19, 20]. These functions provide a plausible mechanistic basis for at least some of the craniofacial, distal limb, and developmental features observed in our cohort. Additional well‐characterized cases will be necessary to refine the phenotype, clarify the full neurologic spectrum, and more precisely define the contribution of USP34 haploinsufficiency to the broader 2p15p16.1 microdeletion phenotype.

## Author Contributions

B.D.W. conceptualized the study. H.W., A.K., B.A.M., N.M., N.O., N.M., P.J.C.K., J.M.V.H., Q.W., J.V.D.K., and B.D.W. collected and analyzed data. H.W. and B.D.W. drafted the manuscript. All authors reviewed, edited, and approved the final version.

## Funding

Funding was provided to the University of Wisconsin Undiagnosed Disease Program by the Department of Pediatrics and the Center for Precision Medicine at the University of Wisconsin School of Medicine and Public Health.

## Conflicts of Interest

The authors declare no conflicts of interest.

## Data Availability

The data that support the findings of this study are available on request from the corresponding author. The data are not publicly available due to privacy or ethical restrictions.

## References

[1] J. Levy , A. Coussement , C. Dupont , et al., “Molecular and Clinical Delineation of 2p15p16.1 Microdeletion Syndrome,” American Journal of Medical Genetics. Part A 173, no. 8 (2017): 2081–2087, 10.1002/ajmg.a.38302. 28573701

[2] H. Bagheri , C. Badduke , Y. Qiao , et al., “Identifying Candidate Genes for 2p15p16.1 Microdeletion Syndrome Using Clinical, Genomic, and Functional Analysis. JCI,” Insight 1, no. 3 (2016): e85461, 10.1172/jci.insight.85461. PMC5033885 27699255

[3] M. Miceli , P. Failla , L. Saccuzzo , et al., “Trait ‐ Driven Analysis of the 2p15p16.1 Microdeletion Syndrome Suggests a Complex Pattern of Interactions Between Candidate Genes,” Genes & Genomics 45, no. 4 (2023): 491–505, 10.1007/s13258-023-01369-7. 36807877 PMC10027778

[4] A. S. E. van Oirsouw , P. Nedbalova , M. Hancarova , et al., “Pathogenic XPO1 Variants Cause a Dominant Neurodevelopmental Disorder,” Genetics in Medicine 27, no. 11 (2025): 101555, 10.1016/j.gim.2025.101555. 40819229

[5] A. Peron , F. D'Arco , K. A. Aldinger , et al., “BCL11A Intellectual Developmental Disorder: Defining the Clinical Spectrum and Genotype‐Phenotype Correlations,” European Journal of Human Genetics 33, no. 3 (2025): 312–324, 10.1038/s41431-024-01701-z. 39448799 PMC11893779

[6] M. Fannemel , T. Baroy , A. Holmgren , et al., “Haploinsufficiency of XPO1 and USP34 by a De Novo 230 kb Deletion in 2p15, in a Patient With Mild Intellectual Disability and Cranio‐Facial Dysmorphisms,” European Journal of Medical Genetics 57, no. 9 (2014): 513–519, 10.1016/j.ejmg.2014.05.008. 24911659

[7] K. Shimojima , N. Okamoto , and T. Yamamoto , “Characteristics of 2p15‐p16.1 Microdeletion Syndrome: Review and Description of Two Additional Patients,” Congenit Anom (Kyoto) 55, no. 3 (2015): 125–132, 10.1111/cga.12112. 25900130

[8] L. Ronzoni , V. Saletti , G. Scuvera , S. Esposito , and D. Milani , “Response to “Characteristics of 2p15‐p16.1 Microdeletion Syndrome: Review and Description of Two Additional Patients”,” Congenit Anom (Kyoto) 55, no. 4 (2015): 191–192, 10.1111/cga.12119. 26153024

[9] X. Song , S. Wang , and L. Li , “New Insights Into the Regulation of Axin Function in Canonical Wnt Signaling Pathway,” Protein & Cell 5, no. 3 (2014): 186–193, 10.1007/s13238-014-0019-2. 24474204 PMC3967064

[10] T. T. Lui , C. Lacroix , S. M. Ahmed , et al., “The Ubiquitin‐Specific Protease USP34 Regulates Axin Stability and Wnt/Beta‐Catenin Signaling,” Molecular and Cellular Biology 31, no. 10 (2011): 2053–2065, 10.1128/MCB.01094-10. 21383061 PMC3133363

[11] M. O. Caracci , M. E. Avila , F. A. Espinoza‐Cavieres , H. R. Lopez , G. D. Ugarte , and G. V. De Ferrari , “Wnt/Beta‐Catenin‐Dependent Transcription in Autism Spectrum Disorders,” Frontiers in Molecular Neuroscience 14 (2021): 764756, 10.3389/fnmol.2021.764756. 34858139 PMC8632544

[12] V. Kwan , B. K. Unda , and K. K. Singh , “Wnt Signaling Networks in Autism Spectrum Disorder and Intellectual Disability,” Journal of Neurodevelopmental Disorders 8 (2016): 45, 10.1186/s11689-016-9176-3. 27980692 PMC5137220

[13] K. J. Karczewski , L. C. Francioli , G. Tiao , et al., “The Mutational Constraint Spectrum Quantified From Variation in 141,456 Humans,” Nature 581, no. 7809 (2020): 434–443, 10.1038/s41586-020-2308-7. 32461654 PMC7334197

[14] N. Sobreira , F. Schiettecatte , D. Valle , and A. Hamosh , “GeneMatcher: A Matching Tool for Connecting Investigators With an Interest in the Same Gene,” Human Mutation 36, no. 10 (2015): 928–930, 10.1002/humu.22844. 26220891 PMC4833888

[15] K. Jaganathan , S. Kyriazopoulou Panagiotopoulou , J. F. McRae , et al., “Predicting Splicing From Primary Sequence With Deep Learning,” Cell 176, no. 3 (2019): 535–48.e24, 10.1016/j.cell.2018.12.015. 30661751

[16] E. Rajcan‐Separovic , C. Harvard , X. Liu , et al., “Clinical and Molecular Cytogenetic Characterisation of a Newly Recognised Microdeletion Syndrome Involving 2p15‐16.1,” Journal of Medical Genetics 44, no. 4 (2007): 269–276, 10.1136/jmg.2006.045013. 16963482 PMC2598046

[17] G. Reka , K. Wojciechowska , and M. Lejman , “Rare Case of De Novo 2p15 Microdeletion Syndrome With Deletion Covering XPO1 and USP34 Genes Diagnosed in a Child ‐ A Case Report,” Application of Clinical Genetics 17 (2024): 117–124, 10.2147/TACG.S465575. 39050773 PMC11268518

[18] G. J. Woodhead , C. A. Mutch , E. C. Olson , and A. Chenn , “Cell‐Autonomous Beta‐Catenin Signaling Regulates Cortical Precursor Proliferation,” Journal of Neuroscience 26, no. 48 (2006): 12620–12630, 10.1523/JNEUROSCI.3180-06.2006. 17135424 PMC2867669

[19] D. Zechner , Y. Fujita , J. Hulsken , et al., “Beta‐Catenin Signals Regulate Cell Growth and the Balance Between Progenitor Cell Expansion and Differentiation in the Nervous System,” Developmental Biology 258, no. 2 (2003): 406–418, 10.1016/s0012-1606(03)00123-4. 12798297

[20] Y. C. Guo , M. Y. Wang , S. W. Zhang , et al., “Ubiquitin‐Specific Protease USP34 Controls Osteogenic Differentiation and Bone Formation by Regulating BMP2 Signaling,” EMBO Journal 37, no. 20 (2018): EMBJ201899398, 10.15252/embj.201899398. PMC6187217 30181118

